# Pre- and Interhospital Workflow Times for Patients With Large Vessel Occlusion Stroke Transferred for Endovasvular Thrombectomy

**DOI:** 10.3389/fneur.2021.730250

**Published:** 2021-08-26

**Authors:** Laura C. C. van Meenen, Frank Riedijk, Jeffrey Stolp, Bas van der Veen, Patricia H. A. Halkes, Taco C. van der Ree, Charles B. L. M. Majoie, Yvo B. W. E. M. Roos, Martin D. Smeekes, Jonathan M. Coutinho

**Affiliations:** ^1^Department of Neurology, Amsterdam UMC, University of Amsterdam, Amsterdam, Netherlands; ^2^Emergency Medical Services North-Holland North, Alkmaar, Netherlands; ^3^Department of Neurology, Noordwest Ziekenhuisgroep, Alkmaar, Netherlands; ^4^Department of Neurology, Dijklander Ziekenhuis, Hoorn, Netherlands; ^5^Department of Radiology and Nuclear Medicine, Amsterdam UMC, University of Amsterdam, Amsterdam, Netherlands

**Keywords:** acute ischemic stroke, large vessel occlusion, prehospital/EMS, interhospital, workflow, time to treatment, endovascular thrombectomy

## Abstract

**Background:** Patients with large vessel occlusion (LVO) stroke are often initially admitted to a primary stroke center (PSC) and subsequently transferred to a comprehensive stroke center (CSC) for endovascular thrombectomy (EVT). This interhospital transfer delays initiation of EVT. To identify potential workflow improvements, we analyzed pre- and interhospital time metrics for patients with LVO stroke who were transferred from a PSC for EVT.

**Methods:** We used data from the regional emergency medical services and our EVT registry. We included patients with LVO stroke who were transferred from three nearby PSCs for EVT (2014–2021). The time interval between first alarm and arrival at the CSC (call-to-CSC time) and other time metrics were calculated. We analyzed associations between various clinical and workflow-related factors and call-to-CSC time, using multivariable linear regression.

**Results:** We included 198 patients with LVO stroke. Mean age was 70 years (±14.9), median baseline NIHSS was 14 (IQR: 9–18), 136/198 (69%) were treated with intravenous thrombolysis, and 135/198 (68%) underwent EVT. Median call-to-CSC time was 162 min (IQR: 137–190). In 133/155 (86%) cases, the ambulance for transfer to the CSC was dispatched with the highest level of urgency. This was associated with shorter call-to-CSC time (adjusted β [95% CI]: −27.6 min [−51.2 to −3.9]). No clinical characteristics were associated with call-to-CSC time.

**Conclusion:** In patients transferred from a PSC for EVT, median call-to-CSC time was over 2.5 h. The highest level of urgency for dispatch of ambulances for EVT transfers should be used, as this clearly decreases time to treatment.

## Introduction

Endovascular treatment (EVT) is routine care for patients with large vessel occlusion (LVO) stroke of the anterior circulation (1, 2). EVT can be performed in specialized hospitals only, so-called comprehensive stroke centers (CSC). In ~45 to 83% of cases (3–6), patients with LVO stroke are first admitted to a primary stroke center (PSC), where they undergo diagnostic evaluation and, if indicated, treatment with intravenous thrombolysis (IVT). Patients who are potentially eligible for EVT are subsequently transferred to a CSC. This “drip-and-ship” model delays initiation of EVT by 40 to 106 min (3, 4). Timely initiation of EVT is of vital importance, because it increases the chance of good clinical outcome (7). Multiple studies have reported in detail on EVT-related time intervals after arrival at the CSC, such as door-to-CT and door-to-groin time (8–11), and innovations to shorten these time intervals have been studied and successfully implemented (9–13). In recent years, several measures to improve the prehospital and interhospital workflow prior to EVT have also been proposed (14–16). However, little is known about the distribution of time intervals before arrival at the CSC. Toward future implementation of measures to decrease treatment delay, we aimed to study the pre- and interhospital time metrics in patients with LVO stroke who were transferred from a PSC to a CSC for EVT.

## Materials and Methods

### Study Design and Population

For this study, we used prehospital and interhospital workflow data that were prospectively collected by emergency medical services (EMS) North-Holland North, the Netherlands. EMS North-Holland North has a catchment area of 1,400 square kilometers with ~650.000 inhabitants. For clinical and in-hospital workflow data, we used the prospective stroke registry of the Amsterdam UMC, the Netherlands. Amsterdam UMC has a catchment area for EVT with approximately 3.3 million inhabitants and receives EVT referrals from 11 nearby PSCs. We included adult patients who had an LVO stroke between January 1 2014 and April 1 2021, who were first transported to one of three PSCs in North-Holland (Northwest Clinics locations Alkmaar and Den Helder, and Dijklander Hospital location Hoorn), and who were subsequently transferred to Amsterdam UMC to potentially undergo EVT. We excluded patients with an in-hospital stroke and patients of whom no EMS data were available.

All patients eligible for inclusion were sent a letter with detailed information about the study. The patient or legal representative had the opportunity to deny permission for use of data via an opt-out form, in accordance with the European Union General Data Protection Regulation and institutional guidelines.

### Definitions, Procedures and Outcomes

Time of symptom onset was defined as the time of witnessed symptom onset or, if this was unknown, the time that the patient was last known to be well. In the Netherlands, for urgent ambulance dispatch, there are two levels of urgency: A1 and A2. The A1-dispatch is used for potentially life threatening situations and the target response time (time between ambulance dispatch and arrival at the patient's location) is 15 min. The A2-dispatch is used for urgent, but non-life threatening situations; the dispatched ambulance aims to arrive at the patient's location within 30 min. The National Institutes of Health Stroke Scale (NIHSS) was used to quantify the severity of neurological deficit on arrival at the PSC. EVT was defined as arterial puncture in the angiography suite, with the objective to perform mechanical thrombectomy with a stent retriever and/or thrombus aspiration.

We defined the following time points within the EVT-related workflow: time of symptom onset, time of first call to the dispatch center, time of first ambulance dispatch, time of first ambulance arrival at the patient's location, time of first ambulance departure from the patient's location, time of first ambulance arrival at the PSC, time of initiation of IVT, time of second call to the dispatch center, time of second ambulance dispatch, time of second ambulance arrival at the PSC, time of second ambulance departure from the PSC, time of second ambulance arrival at the CSC, and time of initiation of EVT (groin puncture).

All consecutive intervals between the different time points were calculated. Our primary workflow measure was the time between the first call to the dispatch center and patient arrival at the CSC (call-to-CSC time). Other outcomes were time between first ambulance arrival at the patient's location and first ambulance departure to the PSC (on-scene time), time between first call to the dispatch center and arrival at the PSC (call-to-PSC time), time between patient arrival at the PSC and time of second ambulance departure from the PSC (door-in-door-out time), and time between second ambulance departure from the PSC and arrival at the CSC (transfer time).

### Statistical Analysis

Baseline characteristics of patients who were excluded because of missing EMS data were compared to those of included patients, using independent samples *t*-test for normally distributed continuous variables, Mann-Whitney *U* test for non-normally distributed continuous variables, and χ^2^ test for categorical variables. For included patients, baseline characteristics were reported for the population as a whole. For all consecutive intervals between the different time points, the median time in minutes was calculated. We used multivariable linear regression to perform an exploratory analysis of the associations between clinical and workflow-related factors and call-to-CSC time, call-to-PSC time, and door-in-door-out time. For our analysis of call-to-CSC time, we used the following variables (unless reported otherwise, baseline characteristics were measured on arrival at the PSC): age, previous ischemic stroke or transient ischemic attack (TIA), baseline systolic blood pressure, baseline diastolic blood pressure, baseline NIHSS, location of occlusion, treatment with IVT, time between symptom onset and first call to dispatch center (onset-to-call time), time of first call to dispatch center (within or outside office hours), person making the first call to the dispatch center (non-medical person or general practitioner), urgency of first ambulance dispatch, and urgency of second ambulance dispatch. When analyzing call-to-PSC time, the following variables were used: age, previous ischemic stroke/TIA, baseline NIHSS, onset-to-call time, time of first call to dispatch center, person making first call to dispatch center, and urgency of first ambulance dispatch. For our analysis of door-in-door-out-time, we used age, previous ischemic stroke/TIA, baseline systolic blood pressure, baseline diastolic blood pressure, baseline NIHSS, location of occlusion, treatment with IVT, time between symptom onset and arrival at the PSC (onset-to-PSC time), time of arrival at the PSC (within or outside office hours), and urgency of second ambulance dispatch. For all regression analyses, we imputed missing values using multiple imputation, using the following variables: age, previous ischemic stroke/TIA, history of hypertension, history of diabetes mellitus, history of atrial fibrillation, pre-stroke modified Rankin Scale score (mRS), baseline systolic blood pressure, baseline diastolic blood pressure, baseline NIHSS, location of occlusion on CTA, treatment with IVT, treatment with EVT, 90-day mRS, time of first call to the dispatch center, person making the first call to the dispatch center, urgency of first ambulance dispatch, number of diagnostic procedures or interventions performed by ambulance paramedics on-scene, distance from patient's location to PSC, time of arrival at PSC, urgency of second ambulance dispatch, distance between PSC and CSC, call-to-CSC time, on-scene time, call-to-PSC time, door-in-door-out time, transfer time, onset-to-call time, onset-to-scene time and onset-to-PSC time. All analyses were be performed using SPSS (version 25; SPSS Inc., Chicago, IL, USA).

## Data Availability Statement

Individual patient data cannot be made available under Dutch law because we did not obtain patient approval for sharing individual patient data, even in coded form. However, all syntax files and output of statistical analyses will be made available upon reasonable request.

## Results

During the study period, 288 patients were transferred from one of the three PSCs to our hospital to assess eligibility for EVT. Of these, 90 patients were excluded because no EMS data were available (*n* = 68), they had an in-hospital stroke (*n* = 16), they objected to use of data (*n* = 5) or they were <18 years old (*n* = 1). Therefore, 198/288 (69%) patients were included in the study (Supplementary Figure 1). Baseline characteristics of patients who were excluded because of missing EMS data did not differ from those of included patients, except for pre-stroke mRS scores, which were slightly lower among the excluded patients [median (IQR): 0 (0-0) vs. 0 (0-1), *p* = 0.01].

Baseline characteristics are reported in Table 1. Included patients had a mean age of 70 (± 14.9), a median baseline NIHSS of 14 (IQR: 9–18), were treated with IVT in 136/198 (69%) and with EVT in 135/198 (68%) cases. The most common reasons for refraining from EVT were dissolution of the LVO upon arrival at the CSC [27/63 (43%)], unfavorable radiological characteristics [9/63 (14%)], and a combination of clinical and radiological characteristics [8/63 (13%)].

**Table 1:** Baseline characteristics.

	**All patients (*n* = 198)**
**Clinical characteristics**	
Age, years—mean ± SD	70 ± 14.9
Sex, male—no./total (%)	94/198 (48%)
Hypertension—no./total (%)	74/196 (38%)
Diabetes mellitus—no./total (%)	31/196 (16%)
Atrial fibrillation—no./total (%)	44/196 (22%)
Previous ischemic stroke/TIA—no./total (%)	36/196 (18%)
Pre-stroke mRS score^a^–median (IQR)	0 (0–1)
Systolic blood pressure on arrival at PSC^b^–mean ± SD	157 ± 27.6
Diastolic blood pressure on arrival at PSC^c^–mean ± SD	89 ± 15.7
NIHSS score on arrival at PSC^d^–median (IQR)	14 (9–18)
Intracranial occlusion site on CTA—no./total (%)	
Intracranial ICA	35/198 (18%)
M1	118/198 (60%)
M2	28/198 (14%)
Basilar artery	11/198 (6%)
Other	6/198 (3%)
Treatment with IVT—no./total (%)	136/198 (69%)
**Workflow-related factors**	
First call to dispatch center outside office hours—no./total (%)	110/167 (66%)
Person making first call to dispatch center—no./total (%)
Non-medical person	103/108 (95%)
General practitioner	5/108 (5%)
Urgency of first ambulance dispatch, A1—no./total (%)	163/167 (98%)
Distance between patient's location and PSC, kilometers^e^–median (IQR)	1 (5–17)
Arrival at PSC outside office hours—no./total (%)	109/167 (65%)
Urgency of second ambulance dispatch, A1—no./total (%)	133/155 (86%)
Distance between PSC and CSC, kilometers—median (IQR)	54 (54–57)

*A1, the A1 ambulance dispatch (most urgent) is used for potentially life threatening situations; target response time is 15 min; CSC, comprehensive stroke center; CTA, computed tomography angiography; EVT, endovascular thrombectomy; ICA, internal carotid artery; IQR, interquartile range; IVT, intravenous thrombolysis; M1, first segment of the middle cerebral artery; M2, second segment of the middle cerebral artery; mRS, modified Rankin Scale; NIHSS, National Institutes of Health Stroke Scale; no., number; PSC, primary stroke center; SD, standard deviation; TIA, transient ischemic attack. Number of missing values*:

a *78*;

b *38*;

c *39*;

d *7*;

e *31*.

The first call to the dispatch center was made by a non-medical person in 103/108 (95%) and by a general practitioner in 5/108 (5%) cases, and was made outside office hours in 110/167 (66%). The urgency of the first ambulance dispatch was A1 in 163/167 (98%), while the urgency of the second ambulance dispatch was A1 in 133/155 (86%) cases. All patients were transported over ground.

**Figure 1:**
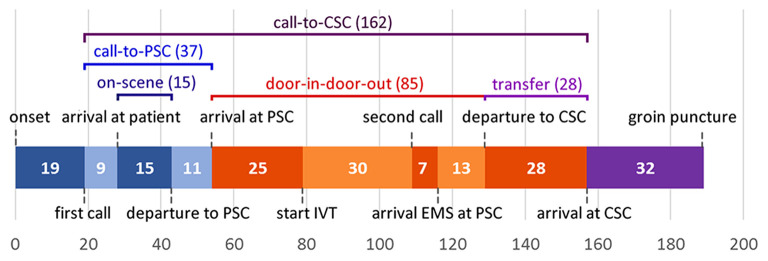
Median time intervals from symptom onset to arrival at the CSC. CSC, comprehensive stroke center; EMS, emergency medical services; IVT, intravenous thrombolysis; PSC, primary stroke center. Not included in figure: median time between first call to dispatch center and first ambulance dispatch: 1 min; median time between second call to dispatch center and second ambulance dispatch: 1 min.

All pre-defined consecutive median time intervals are shown in Figure 1. Median call-to-CSC time was 162 min (IQR 137–190). Median on-scene time was 15 min (IQR 11–20), call-to-PSC time 37 min (IQR 29–45), door-in-door-out time 85 min (IQR 70–113) and transfer time 28 min (IQR 26–30).

The following factors were associated with call-to-CSC time in univariable analyses: baseline systolic blood pressure [unadjusted β (95% CI): 0.5 min (0.2 to 0.8)], baseline diastolic blood pressure [unadjusted β (95% CI): 0.8 min (0.1 to 1.5)], baseline NIHSS [unadjusted β (95% CI): −1.4 min (−2.8 to −0.8)] and the person making the first call to the dispatch center [unadjusted β for general practitioner (95% CI): 25.8 min (4.7 to 46.8)]. In the multivariable model, only two factors were associated with call-to-CSC time: the person making the first call to dispatch center [adjusted β for general practitioner (95% CI): 34.2 min (7.2 to 61.1)] and urgency level of dispatch of the transferring ambulance [adjusted β for A1 (95% CI): −27.6 min (−51.2 to−3.9); Table 2]. Call-to-PSC time was only associated with onset-to-call time [adjusted β for every 10-min increase (95% CI): 0.1 min (0.04 to 0.2); Supplementary Table 1] and door-in-door-out time was associated with urgency level of dispatch of the transferring ambulance [adjusted β for A1 (95% CI): −30.0 min (−56.4 to −3.7); Supplementary Table 2].

**Table 2:** Clinical and workflow-related factors associated with call-to-CSC time.

	**Univariable model—unadjusted β in minutes (95% CI)**	**Multivariable model—adjusted β in minutes (95% CI)**
**Clinical factors**		
Age	−0.1 (−0.7 to 0.5)	−0.2 (−0.9 to 0.5)
Previous acute ischemic stroke/TIA^a^	−23.1 (−46.5 to 0.4)	−17.0 (−40.8 to 6.8)
Systolic blood pressure on arrival at PSC^b^	0.5 (0.2 to 0.8)	0.3 (−0.1 to 0.8)
Diastolic blood pressure on arrival at PSC^c^	0.8 (0.1 to 1.5)	0.5 (−0.2 to 1.2)
NIHSS on arrival at PSC^d^	−1.4 (−2.8 to −0.8)	−1.0 (−2.4 to 0.4)
Location of occlusion, anterior circulation^e^	−9.5 (−42.0 to 23.1)	−1.1 (−35.0 to 32.9)
Treatment with IVT	−9.2 (−29.0 to 10.7)	6.2 (−17.3 to 29.8)
**Workflow-related factors**		
Onset-to-call time^f^	0.3 (−0.2 to 0.8)	0.4 (−0.2 to 1.0)
First call to dispatch center outside office hours^g^	10.8 (−8.9 to 30.4)	11.1 (−10.0 to 32.3)
Person making first call to dispatch center, general practitioner^h^	25.8 (4.7 to 46.8)	34.2 (7.2 to 61.1)
Urgency of first ambulance dispatch, A1^i^	−0.7 (−0.3 to 31.0)	−2.5 (−45.2 to 40.2)
Urgency of second ambulance dispatch, A1^j^	−24.7 (−50.6 to 1.3)	−27.6 (−51.2 to −3.9)

*A1, the A1 ambulance dispatch (most urgent) is used for potentially life threatening situations; target response time is 15 min; call-to-CSC time, time between first call to the dispatch center and arrival at the CSC; CI, confidence interval; CSC, comprehensive stroke center; IVT, intravenous thrombolysis; NIHSS, National Institutes of Health Stroke Scale; onset-to-call time, time between symptom onset and first call to the dispatch center; PSC, primary stroke center; TIA, transient ischemic attack*.

*Number of missing values*:

a *2*;

b *38*;

c *39*;

d *7*;

f *35*;

g *31*;

h *90*;

i *31*;

j *43; call-to-CSC time: 75*.

e *Intracranial part of internal carotid artery, first of middle cerebral artery (M1), or second segment of middle cerebral artery*.

*^f^β is reported per 10-min increase in onset-to-call time*.

## Discussion

In this cohort study of patients transferred from a PSC for EVT in the Netherlands, median call-to-CSC time was more than 2.5 h. We found that dispatching the transferring ambulance with the highest level of urgency was associated with a 28-min decrease in time to arrival at the CSC. If the first call to the dispatch center was made by a general practitioner, this was associated with a delay of 34 min, although this was the case for only 5% of patients. Clinical characteristics were not independently associated with any of the prehospital or interhospital time intervals.

Ever since EVT has become standard care for patients with LVO stroke and its effect has been shown to be highly time-dependent (7), many studies have examined measures to improve EVT-related logistics inside the CSC, leading to a fairly streamlined in-hospital workflow (8–13). However, the workflow prior to arrival at the CSC—pre- and interhospital workflow—has only recently started to gain attention (14–16), and is currently considered one of the main “bottlenecks” in acute stroke management (17, 18). Few previous studies have reported in detail on time metrics prior to arrival at the CSC. However, in the field of acute myocardial infarction, which deals with logistical challenges similar to stroke regarding transportation of patients to hospitals capable of percutaneous coronary intervention (PCI), pre- and interhospital time intervals are regularly reported. It is noteworthy that in this field, compared to that of acute ischemic stroke, time metrics are generally substantially shorter. The average time between first alarm and initiation of PCI (call-to-balloon time) for patients who are transferred from a non-PCI-capable hospital is 143–160 min (19–21). Taking into account that this includes time between arrival at the intervention center and initiation of PCI, which is around 36–43 min on average (20, 22), time from first alarm to arrival at the intervention center in these studies is significantly shorter than our call-to-CSC time of 162 min. Our door-in-door-out times, which were very similar to those reported in previous literature (23, 24), were also substantially longer than those found in interventional cardiology studies: 85 vs. 52 min (21). Although these differences may be partly explained by substantive differences between myocardial infarction and acute ischemic stroke, for example, regarding diagnostic procedures and treatment, it seems as though pre- and interhospital logistics are more optimally streamlined in the field of interventional cardiology than in that of acute ischemic stroke. Further research may focus on identifying potential measures for improvement of the EVT workflow that have been shown to decrease pre- and interhospital delay in patients being transferred to undergo PCI.

Our finding that in 14% of patients the transferring ambulance was not dispatched with the highest level of urgency is somewhat surprising. Although the nationwide protocol for ambulance care in the Netherlands does not mention a recommended level of urgency for dispatch of ambulances transferring patients for EVT (25), both regional and national stroke care protocols state that ambulances for EVT transfers should be requested with the highest level of urgency (26, 27). In other countries, stroke care protocols differ in their recommendations. The National Stroke Service Model of the NHS (United Kingdom) recommends that interhospital transfers for EVT should be treated at least as a category 2 call; this is the second highest level of urgency for ambulance dispatch, for which the response target time is 18 min (28). The American Stroke Association has stated that stroke warrants a priority ambulance dispatch and that rapid transfer of stroke patients for EVT should be ensured, but has not made recommendations regarding the urgency with which transferring ambulances should be dispatched (29, 30). The necessity of dispatching ambulances for EVT transfer with the highest level of urgency needs to be conveyed to EMS and dispatch organizations, as well as referring PSCs. Incorporating a recommended highest level of urgency for dispatch of ambulances for EVT transfers into stroke care protocols should be considered, as this clearly decreases door-in-door-out time and overall call-to-CSC time.

The association between the general practitioner making the first call to the dispatch center and longer call-to-CSC time may be explained by patients with mild or fluctuating neurological deficits potentially being inclined to visit a general practitioner first, while (bystanders of) patients with evident, severe neurological deficits may be more likely to directly contact the dispatch center. Because patients with mild or fluctuating neurological deficits may be more difficult to diagnose or may be observed for a longer time before transfer to a CSC is initiated, call-to-CSC time may be longer in these patients. Nonetheless, since contacting a general practitioner first is associated with substantially longer call-to-CSC times, and may even cause further delay prior to the first call to the dispatch center, efforts should be taken to promote directly contacting the local emergency phone number in case of symptoms of a potential stroke. Considering that only 5% of calls to the dispatch center were made by a general practitioner in our cohort, this does not seem to be a major contributing factor to prehospital treatment delay in the Netherlands.

Finally, it should be noted that our median time between initiation of IVT and the second call to the dispatch center was 30 min, which seems fairly long considering that acquisition of acute neuroimaging is often completed prior to initiation of IVT. Factors that may contribute to delay within this time interval are acquisition and/or assessment of CT angiography after initiation of IVT, evaluation of the clinical response to IVT before initiating transfer, and assessment of neuroimaging by a CSC radiologist prior to initiating transfer. According to several guidelines, CT angiography should be acquired—in patients who are potentially eligible for EVT—either prior to or immediately after initiation of IVT. Furthermore, patients should not be observed for assessment of clinical response to IVT prior to initiating the process of transfer to a CSC (26, 27, 29). However, it is unknown how often these guidelines are adhered to in clinical practice. When it comes to forwarding neuroimaging to the CSC, technical issues may be a cause of delay (24). Therefore, a fast and reliable system for forwarding imaging should be implemented, and—in straight-forward cases—requesting the transferring ambulance prior to receiving definitive approval by the CSC may be considered.

There are two important limitations to this study. First, data collection for this study took place in the Netherlands, where ambulance care is provided by the government in partnership with private organizations and is coordinated by overarching dispatch centers. Furthermore, it is a densely populated country where hospitals are located relatively close to one another. The Netherlands also has an overall good road and highway infrastructure, which makes even remote hospitals relatively easy to reach. In a recent cohort study of patients transferred for EVT in the USA (31), average time between first call to the dispatch center and arrival of EMS at the patient's location when traveling over ground was 16 min, compared to 7 min in our study. Average travel distance between PSC and CSC was 47 miles, resulting in a transfer time of 50 min, while in our study the median travel distance was 54 kilometers (33 miles), with a median transfer time of 28 min. Because ambulance travel times in the Netherlands are relatively short, our findings should be extrapolated to other countries with caution. The second limitation to this study is that we had relatively high numbers of missing data for some variables. Because EMS data could not be retrieved in 90/288 (31%) patients who were transferred for EVT during the study period, these patients were excluded from the study. Among the included patients, we had high numbers of missing values for the person making the first call to the dispatch center (45%), call-to-CSC time (38%) and door-in-door-out time (38%). The high numbers of missing EMS data may be due to the emergency setting and population in which these data were collected—patients with a (suspected) stroke in need of urgent care—potentially leading to time constraints when it comes to administrative duties. In order to check for selection bias as a result of the exclusion of patients with no available EMS data, we compared baseline characteristics of included patients to those of patients who were excluded because EMS data were not available. Since baseline characteristics did not differ between groups, except for slightly lower pre-stroke mRS scores among the excluded patients, we did not find any indication of selection bias in this regard. To try to reduce the impact of the missing values on our analyses, we used multiple imputation.

In conclusion, in patients transferred from a PSC for EVT in the Netherlands, median call-to-CSC time was 162 min. If the first call to the dispatch center was made by a general practitioner, this was associated with a delay of 34 min, although this was the case for only 5% of patients. Dispatching the ambulance for transfer to the CSC with the highest level of urgency was associated with a 28-min decrease in call-to-CSC time. The general population should be instructed to contact the local emergency phone number directly in case of stroke symptoms, and incorporating a recommended level of urgency for dispatch of ambulances for EVT transfers into stroke care protocols should be considered.

## Data Availability Statement

Individual patient data cannot be made available under Dutch law because we did not obtain patient approval for sharing individual patient data, even in coded form. However, all syntax files and output of statistical analyses will be made available upon reasonable request. Requests to access the datasets should be directed to j.coutinho@amsterdamumc.nl.

## Ethics Statement

Ethical review and approval was not required for the study on human participants in accordance with the local legislation and institutional requirements. Written informed consent for participation was not required for this study in accordance with the national legislation and the institutional requirements.

## Author Contributions

Data collection and analysis was performed by LM, FR, and JC. The first draft of the manuscript was written by LM. All authors contributed to the study conception and design, commented on previous versions of the manuscript, read, and approved the final manuscript.

## Conflict of Interest

CM reports grants from CVON/Dutch Heart Foundation, European Commission, TWIN Foundation, Stryker, and Health Evaluation Netherlands, all outside the submitted work (paid to institution), and is shareholder of Nico.lab, a company that focuses on the use of artificial intelligence for medical image analysis. YR is a minor shareholder of Nico.lab. JC received unrelated research support from the Dutch Heart Foundation, Bayer, Boehringer, and Medtronic. All fees were paid to his employer. The remaining authors declare that the research was conducted in the absence of any commercial or financial relationships that could be construed as a potential conflict of interest.

## Publisher's Note

All claims expressed in this article are solely those of the authors and do not necessarily represent those of their affiliated organizations, or those of the publisher, the editors and the reviewers. Any product that may be evaluated in this article, or claim that may be made by its manufacturer, is not guaranteed or endorsed by the publisher.
