# Supplementary material for: Pre- and Interhospital Workflow Times for Patients With Large Vessel Occlusion Stroke Transferred for Endovasvular Thrombectomy
Source: Front Neurol. 2021 Aug 26;12:730250. doi: 10.3389/fneur.2021.730250 (PMC8428365; doi:10.3389/fneur.2021.730250)
Supplement: Supplementary file 1 [file Data_Sheet_1.docx]

Supplementary Material

**Supplementary table 1. Clinical and workflow-related factors associated with call-to-PSC time**

|  | **Univariable model – unadjusted β in minutes (95% CI)** | **Multivariable model – adjusted β in minutes (95% CI)** |
| --- | --- | --- |
| **Clinical factors** |  |  |
| Age | -0.1 (-0.2 to 0.1) | -0.04 (-0.2 to 0.1) |
| Previous acute ischemic stroke/TIA^1^ | -2.3 (-7.5 to 2.9) | -2.2 (-7.3 to 3.0) |
| NIHSS on arrival at PSC^2^ | 0.1 (-0.2 to 0.5) | 0.2 (-0.2 to 0.5) |
| **Workflow-related factors** |  |  |
| Onset-to-call time^3^ | 0.1 (0.1 to 0.2) | 0.1 (0.04 to 0.2) |
| First call to dispatch center outside office hours^4^ | 3.5 (-2.2 to 9.2) | 3.3 (-1.2 to 7.9) |
| Person making first call to dispatch center, general practitioner^5^ | -1.5 (-12.3 to 9.3) | -0.6 (-9.4 to 8.2) |
| Urgency of first ambulance dispatch, A1^6^ | -3.1 (-14.6 to 8.4) | -2.4 (-13.8 to 9.1) |

A1 = the A1 ambulance dispatch (most urgent) is used for potentially life threatening situations; target response time is 15 minutes; call-to-PSC time = time between first call to dispatch center and arrival at the PSC; CI = confidence interval; NIHSS = National Institutes of Health Stroke Scale; onset-to-call time = time between symptom onset and first call to dispatch center; PSC = primary stroke center; TIA = transient ischemic attack.

Number of missing values: ^1^2; ^2^7; ^3^35; ^4^31; ^5^90; ^6^31; call-to-PSC time: 32.

^3^β is reported per 10-minute increase in onset-to-call time.

**Supplementary table 2. Clinical and workflow-related factors associated with door-in-door-out time**

|  | **Univariable model – unadjusted β in minutes (95% CI)** | **Multivariable model – adjusted β in minutes (95% CI)** |
| --- | --- | --- |
| **Clinical factors** |  |  |
| Age | -0.1 (-0.7 to 0.6) | -0.3 (-1.0 to 0.4) |
| Previous acute ischemic stroke/TIA^1^ | -19.8 (-44.5 to 4.8) | -16.3 (-41.5 to 8.8) |
| Systolic blood pressure on arrival at PSC^2^ | 0.5 (0.1 to 0.8) | 0.4 (-0.1 to 0.9) |
| Diastolic blood pressure on arrival at PSC^3^ | 0.8 (0.2 to 1.4) | 0.4 (-0.3 to 1.2) |
| NIHSS on arrival at PSC^4^ | -1.4 (-2.8 to -0.01) | -0.9 (-2.4 to 0.6) |
| Location of occlusion, anterior circulation | -10.2 (-43.8 to 23.3) | -0.4 (-35.3 to 34.4) |
| Treatment with IVT | -10.5 (-30.8 to 9.9) | 3.3 (-22.2 to 28.8) |
| **Workflow-related factors** |  |  |
| Onset-to-PSC time^5^ | 0.2 (-0.3 to 0.6) | 0.2 (-0.4 to 0.7) |
| Arrival at PSC outside office hours^6^ | 7.2 (-12.0 to 26.4) | 0.7 (-19.6 to 21.1) |
| Urgency of second ambulance dispatch, A1^7^ | -30.1 (-56.7 to -3.4) | -30.0 (-56.4 to -3.7) |

A1 = the A1 ambulance dispatch (most urgent) is used for potentially life threatening situations; target response time is 15 minutes; CI = confidence interval; door-in-door-out time = time between patient arrival at the PSC and time of second ambulance departure from the PSC; ECG = electrocardiography; IVT = intravenous thrombolysis; mRS = modified Rankin Scale; NIHSS = National Institutes of Health Stroke Scale; onset-to-PSC time = time between symptom onset and arrival at PSC; PSC = primary stroke center; TIA = transient ischemic attack.

Number of missing values: ^1^2; ^2^38; ^3^39; ^4^7; ^5^36; ^6^31; ^7^43; door-in-door-out time: 76.

^5^β is reported per 10-minute increase in onset-to-PSC time

**Supplementary figure 1. Inclusion flow chart.** CSC = comprehensive stroke center; EMS = emergency medical services; EVT = endovascular thrombectomy; LVO = large vessel occlusion; PSC = primary stroke center.
